# Differential Regulation of Adipokines May Influence Migratory Behavior in the White-Throated Sparrow (*Zonotrichia albicollis*)

**DOI:** 10.1371/journal.pone.0059097

**Published:** 2013-06-13

**Authors:** Erica F. Stuber, Jessica Verpeut, Maria Horvat-Gordon, Ramesh Ramachandran, Paul A. Bartell

**Affiliations:** 1 Department of Animal Science, The Pennsylvania State University, University Park, Pennsylvania, United States of America; 2 Ecology Graduate Program, The Pennsylvania State University, University Park, Pennsylvania, United States of America; Liverpool John Moores University, United Kingdom

## Abstract

White-throated sparrows increase fat deposits during pre-migratory periods and rely on these fat stores to fuel migration. Adipose tissue produces hormones and signaling factors in a rhythmic fashion and may be controlled by a clock in adipose tissue or driven by a master clock in the brain. The master clock may convey photoperiodic information from the environment to adipose tissue to facilitate pre-migratory fattening, and adipose tissue may, in turn, release adipokines to indicate the extent of fat energy stores. Here, we present evidence that a change in signal from the adipokines adiponectin and visfatin may act to indicate body condition, thereby influencing an individual's decision to commence migratory flight, or to delay until adequate fat stores are acquired. We quantified plasma adiponectin and visfatin levels across the day in captive birds held under constant photoperiod. The circadian profiles of plasma adiponectin in non-migrating birds were approximately inverse the profiles from migrating birds. Adiponectin levels were positively correlated to body fat, and body fat was inversely related to the appearance of nocturnal migratory restlessness. Visfatin levels were constant across the day and did not correlate with fat deposits; however, a reduction in plasma visfatin concentration occurred during the migratory period. The data suggest that a significant change in the biological control of adipokine expression exists between the two migratory conditions and we propose a role for adiponectin, visfatin and adipose clocks in the regulation of migratory behaviors.

## Introduction

Small songbirds have evolved many strategies to cope with the costly rigors of long-distance migration. During the pre-migratory period, physiological and endocrine systems change in preparation for migration; birds become hyperphagic and alter their metabolism resulting in increased fat deposition [1], [2]. Fat provides more than eight times the energy per gram as protein or carbohydrates and is stored with minimal water, making it a valuable source of energy during migration [3]. The migratory programs of many small songbirds consist of segments of nocturnal flights and subsequent daytime stops to rest and refuel. Flights during successive nights under poor conditions may lower fuel reserves below critical thresholds, requiring birds to delay longer at stopover sites to replenish fat stores [4], [5]. Although many small passerines commence migratory flight after peak fat deposition has been reached [1], how each individual determines whether it has enough fat accumulated to initiate migratory flight is unclear [6], [7]. Pre-migratory hyperphagia and fattening may lead to changes in metabolic signals, such as adipokines, that circulate in the blood of individual birds. Consequently, changes in metabolic signals could be utilized to initiate migration once fat stores reach a critical level. Changes in food intake and dietary composition during pre-migration and the migratory period during stopovers can elicit changes in individuals' migratory behaviors [7]. Birds are able to compensate for low diet quality by increasing food intake and decreasing migratory restlessness [7], [8]. Additionally, previous work has demonstrated that physical condition is related to the initiation of spring migration, where birds in better physical condition leave the wintering site earlier than individuals in worse condition [9], [10]. Birds are able to adjust their behavior in response to changing energetic conditions [11]. Recently, Cerasale et al. [12] have highlighted a potential role of fat hormones in regulating energy intake in migrating birds. However, although a relationship between body condition and migratory behavior has been highlighted in the literature, little is known of the physiological mechanisms linking endogenous information about fat stores and behavioral flexibility.

Avian migrants rely heavily on circannual and circadian clocks to regulate components of migration and the processes of these clocks are shaped by changes in photoperiod [7]. Biological clocks and photoperiodic timers dictate the initiation, duration, termination, and organization of the migratory period (see reviews in [13], [14], [15], [16], [17]). Hyperphagia and pre-migratory fattening are important aspects of the physiological preparation for long-distance flight and these preparations are all under the control of biological clocks [1], [18], [19]. The circannual rhythm of migration is shaped by changes in photoperiod, indicating an influence of environmental cues in regulating this program. The degree to which photoperiodic cues directly regulate migratory behaviors changes among taxa and vary even within the same genus [20]. The appearance of “nocturnal migratory restlessness”, commonly called Zugunruhe, in otherwise diurnal animals is under the direct control of an endogenous circadian clock [21], [22]. In *Zonotrichia albicollis*, the white-throated sparrow (WTSP), it has been demonstrated that circadian clocks and photoperiodic timers regulate the seasonal appearance of Zugunruhe [20], [23], [24]. Clocks are generated by molecular rhythms in the expression and activity of so called “clock genes” which regulate behavioral rhythms through positive and negative autoregulatory feedback loops [25]. Molecular clocks are influenced by metabolism, in particular through alterations in redox states and gas responsive elements [26], [27], [28]. The molecular oscillations of endogenous clocks convey rhythmic information to peripheral systems and modulate system-specific rhythmic behaviors [29]. During the course of the day, rates of lipogenesis and lipolysis vary to accommodate changes in energy demands [30]. Recent evidence demonstrates the existence of molecular circadian clocks located within adipose tissue [31], [32]. Clocks in adipose tissue can be influenced by feeding regimens or central pacemakers through multiple modalities [33] and may precipitate changes in sensitivity to factors promoting either storage or mobilization of triglycerides in anticipation of feeding, fasting, or exercise [34]. Alteration or uncoupling of the clock in adipose tissue from clocks in the brain could arise from changes in the hormonal signals released by adipose cells, such as adiponectin, thereby permitting expression of seasonal behaviors such as hyperphagia or migration.

Although still controversial, many scientists are of the opinion that birds do not produce leptin, a fat hormone with the primary role in mammals of signaling energy sufficiency and adiposity, even though avian leptin receptors are conserved [34], [35], [36]. In non-avian organisms, leptin, which circulates in the blood with a circadian rhythmicity [37], [38], [39], acts on the brain to regulate appetite and feeding behavior [40], metabolism and energy homeostasis, and satiation responses during feeding [41], [42]. Whereas in mammals, adiponectin generally displays a negative correlation with body fat, a positive correlation exists between the amount of circulating leptin and the extent of body fat in mammals [43]. Thus, regulation of energy expenditure via a feedback loop between adiponectin and leptin has been suggested where leptin should decrease food intake (a signal of satiety) and adiponectin should increase food intake (a signal of starvation) [44]. In rodents, leptin stimulates AMP-kinase activity, promoting an increase in fatty acid oxidation [45]. Although leptin receptors have been conserved in avian species, and injections of exogenous leptin induce behavioral responses [46], [47], the DNA which encodes leptin has been lost from the avian genome [36], [46]. A recent study of leptin in migrating birds revealed that birds administered exogenous leptin increased their energy intake [12], but was unable to demonstrate an effect of leptin administration on other key biochemical responses, including downregulation of fatty acid transporter gene expression and increases in the activity of key enzymes involved in fatty acid oxidadtion [48], that are typically observed in mammals. Without endogenously produced leptin, birds must utilize other physiological signals to regulate weight and energy expenditure, as energy homeostasis is critical to successful migration. Adiponectin may subserve the role of leptin in its absence in birds, as it has been hypothesized to act on the brain and with peripheral tissues to mediate ingestive behaviors and energy homeostasis. Consequently, by controlling physiological changes associated with hyperphagia and increased fat deposition, biological clocks may modulate signals of body condition that could inform an individual whether it has enough fuel stored to commence or continue migration.

Historically, adipose tissue was thought to function exclusively as a storage depot for energy. More recently, the role of adipose tissue as an endocrine organ secreting adipokines has been detailed [49], [50], [51]. Adiponectin (ADP), one such adipokine, is a hormone secreted by adipocytes and regulates energy homeostasis [52], [53]. Consequently, adiponectin may play a role in the switch to lipid metabolism as the main source of energy fueling migratory flight in birds. ADP is a self-aggregating protein that circulates in the plasma as trimer, hexamer and (primarily) as a high molecular weight isoform (HMW) [53], [54]. The function of specific isoforms remains unclear, as studies have produced conflicting results [52], [55], [57]. It is generally accepted, however, that HMW isoforms of ADP are considered to be the most physiologically protective for their anti-atherogenic and anti-inflammatory properties [56], [57], [58], [59]. Both normal-weight human subjects and rodent models display circadian rhythmicity of plasma ADP levels and ADP gene expression and plasma ADP concentrations increase after weight loss [19], [60], [61]. Calvani et al. [60] also observed that the daily expression of plasma ADP in obese subjects had smaller amplitudes compared with normal weight subjects. Although ADP is secreted primarily by adipose tissue, ADP and ADP receptor mRNA are also expressed in various other tissues including the liver, skeletal muscle, kidney, pituitary gland, and diencephalon, suggesting autocrine or paracrine roles for this hormone [62], [63]. Because of its role in stimulating fatty-acid oxidation [51], [64], ADP could potentially act as a signal of body condition during the migratory period, a time when birds rely on fat as their main energy source. Adiponectin could signal the brain to modulate energy expenditures during food intake and fasting. We hypothesize that ADP levels are regulated by a biological clock, similar to other autocrine factors such as prolactin [65], serotonin [66], and melatonin [67], [68]. Furthermore, we hypothesize that a change in the circadian rhythm of ADP signaling, whether in amplitude or phase, between non-migratory and migratory conditions could influence migratory activity by communicating changes in adiposity to the clock in the brain which regulates Zugunruhe.

Another potentially important adipokine, visfatin, has been implicated in directly linking metabolism to the molecular clockwork. Few studies have examined avian visfatin. Work performed in chicken has demonstrated that visfatin is expressed to a greater degree in skeletal muscle compared with visceral fat [69]. As a rate-limiting enzyme in the NAD+ biosynthetic pathway, visfatin maintains a reservoir of NAD to function in redox reactions, metabolism, and to act as a substrate for sirtuins [70], [71]. In order to function, SIRT1 requires NAD, whose availability fluctuates with metabolic state [72]. In mammals, SIRT1binds directly to the CLOCK/BMAL dimer complex and is necessary for transcription of core clock genes in the generation of circadian rhythms [26], [73]. Thus, visfatin plays an indirect role in providing metabolic information to biological clocks by regulating the production of NAD [72]. Changes in the biological clockwork of nocturnal migrants occurs during migration [22]; changes in visfatin signals between the non-migratory and migratory periods would provide different information about metabolic state and potentially influence clock mediated migratory behavior. Although controversial, visfatin, which currently has no known receptors, has also been labeled an insulin mimic [74].

**Figure 1:**
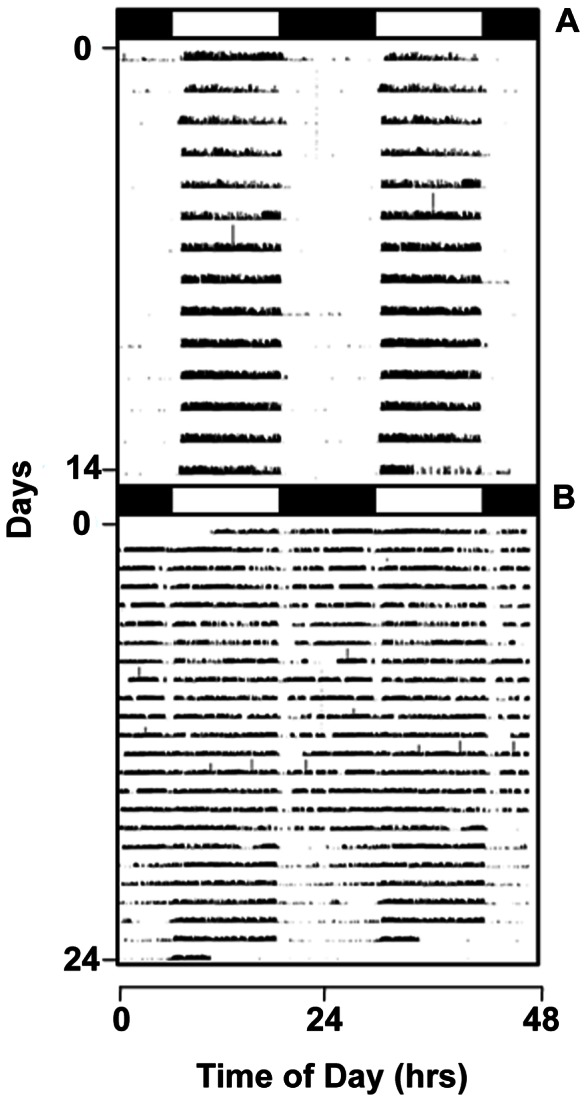
Locomotor activity of non-migrating and migrating white-throated sparrows. Double-plotted actograms displaying locomotor activity recorded from representative non-migrating (**A**) and migrating (**B**) white-throated sparrows under a constant 12 hr light and 12 hr dark photoperiod. White and black bars above each actogram represent hours of lights-on and lights-off, respectively. WTSP are diurnal during the non-migratory period; Zugunruhe was directly observed in migrating individuals using infrared videography during hours of lights-off.

We used Western Blotting and enzyme-linked immunosorbant assay techniques to quantify the levels of ADP and visfatin, respectively, in the blood plasma of a common migratory bird, the white-throated sparrow, *Zonotrichia albicollis*, and compared plasma levels of these adipokines at different times of the day and during the migratory and non-migratory periods in order to determine if migratory behavior occurs concomitant with changes in adipokine signaling. Because white-throated sparrows migrate at night, migratory behavior could be quantified by measuring the amount of nocturnal activity present in individuals while caged (Figure 1). Nocturnal migratory restlessness in white-throated sparrows is under the direct control of a circadian clock [23], [24]. Unlike migratory behaviors in other *Zonotrichia* species, nocturnal migratory restlessness in WTSP is regulated by biological clocks, photoperiod, and the changes in daily expression of locomotor activity [2], [20], [23], [24], [75], [76]. Our results provide new evidence regarding the roles of adipose signaling in birds and that adipose signaling may be used to regulate circannual and circadian migratory behaviors.

## Results

### Detection of plasma adiponectin isoforms

**Figure 2:**
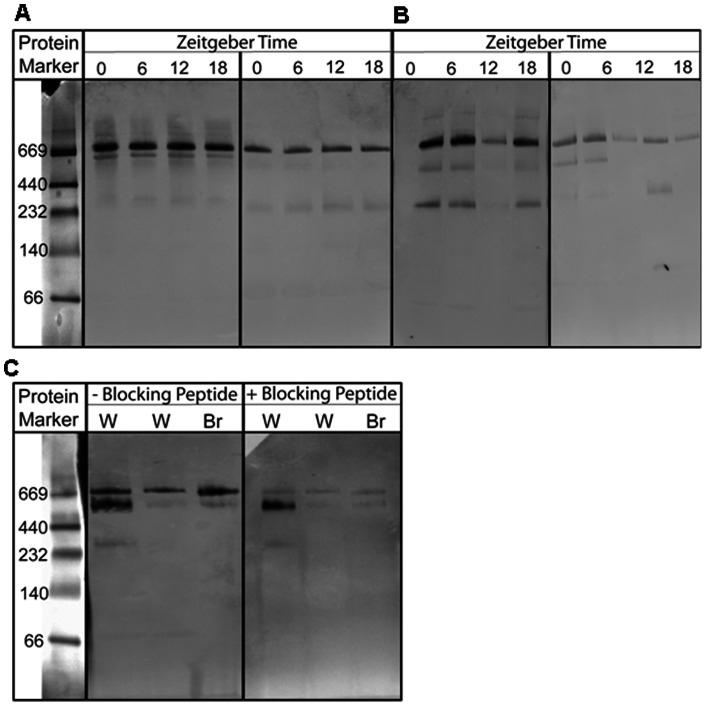
Multiple isoforms of white-throated sparrow plasma adiponectin. Western blot analysis of plasma ADP under native conditions. Representative western blots from two white-throated sparrows **A,** female sparrow in non-migrating condition in the left panel; same bird under migrating conditions in the right panel where plasma samples were collected at four times of day (ZT 0, 6, 12, 18). **B,** Male non-migrating condition in the left panel; same bird under migrating conditions in the right panel where plasma samples were collected at four times of day (ZT 0, 6, 12, 18). For all birds, lights came on at ZT0 and went out at ZT 12. **C,** white-throated sparrow (W) and broiler chicken (Br) plasma separated under native conditions. Primary anti-chicken antibody was either not preadsorbed (− blocking peptide), or preadsorbed with adiponectin peptide (+ blocking peptide). A high molecular weight protein marker was included to determine molecular weight (kDa) of adiponectin isoforms.

To detect multiple isoforms of ADP in white-throated sparrow plasma, we performed Western blot analysis under native (i.e. non-denatured) conditions (Fig. 2A & 2B). Strong immunoreactive bands were detected in sparrow plasma at approximately 720 kDa (which we refer to as HMW), regardless of migratory status and sex. This finding is consistent with the molecular weight and intensity of HMW bands formed in chicken [77]. Weaker bands of approximately 330 kDa were also detected regardless of migratory status and sex. Bands of same molecular weights were previously validated as adiponectin in chicken plasma [77]. All females displayed bands of approximately 540 kDa. Additionally, other trace bands at approximately 67, 136, 450, 670, and 750 kDa were occasionally observed. Multiple isoforms of ADP were observed in the white-throated sparrow, not all of which have been observed in the domestic chicken; although, multimeric bands of similar size were observed in Japanese quail (*Coturnix japonica*) (data not shown). Preadsorption of anti-chicken adiponectin antibody to chicken adiponectin peptide reduced immunostaining of 720 and 540 kDa bands, and eliminated immunostaining of other bands (Fig. 2C) except for bands of 330 kDa which were removed from analysis. All parametric statistics were performed on normalized, log transformed total (sum intensity of all individual isoforms), log transformed HMW ADP, and log transformed visfatin which met the assumptions of normality. Non-parametric statistics were used to analyze normalized molecular weight bands of 67, 136, 450, 540, 670, and 750 kDa ADP, as their expression levels were not normally distributed.

### Rhythmic expression of adiponectin

**Figure 3:**
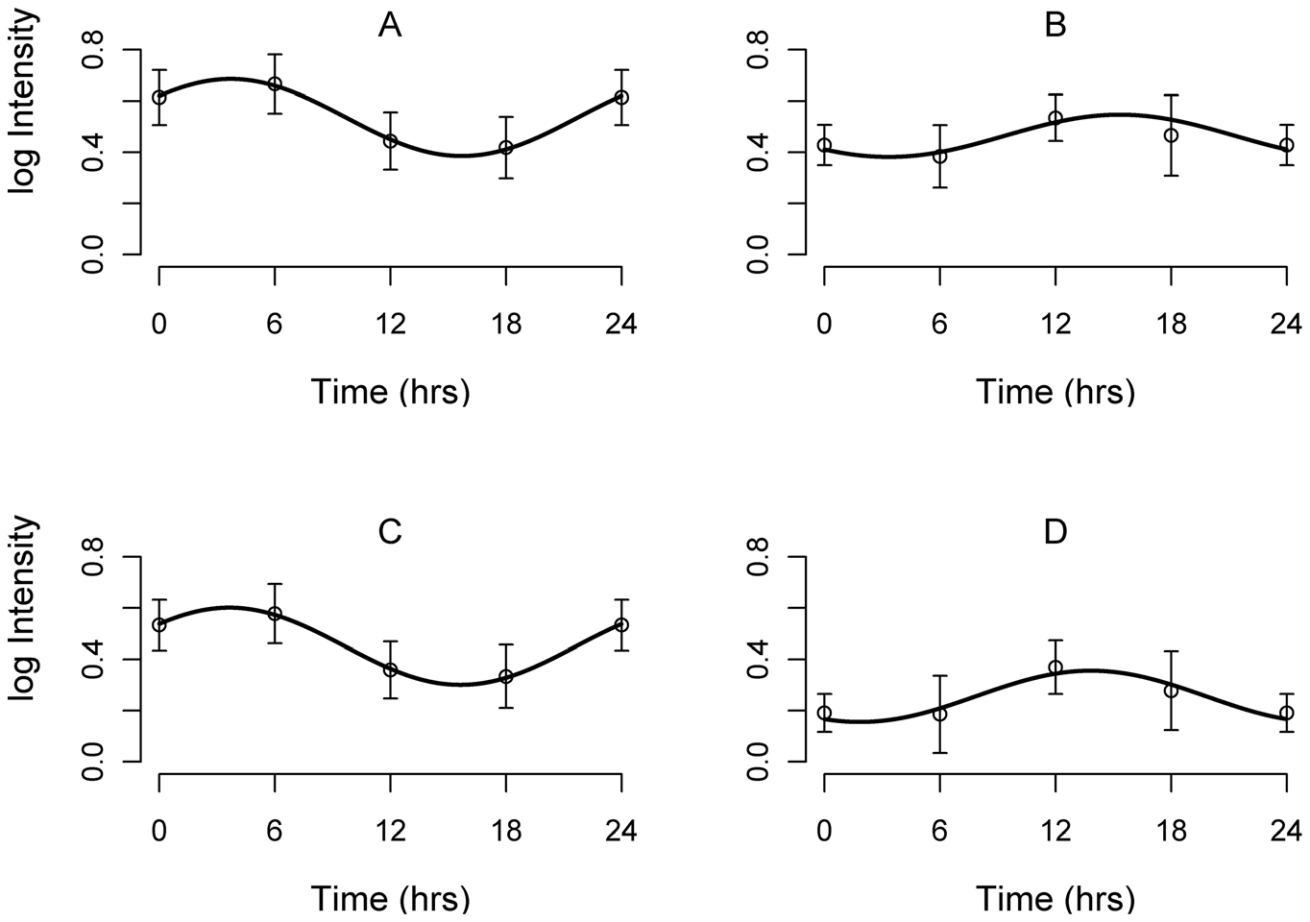
Circadian expression of adiponectin. Cosinor analysis of (**A**) total plasma ADP in non-migrating birds, (**B**) total plasma ADP in migrating birds, (**C**) HMW plasma ADP in non-migrating birds, and (**D**) HMW plasma ADP in migrating birds. For all birds, lights came on at ZT0 and went out at ZT 12. The solid wave shows the best-fit curve determined by linear harmonic regression. Individual data points are plotted as mean ± SE of actual measurements. ZT0 has been double-plotted as ZT24 for visualization.

Using cosinor analysis, we determined that non-migrating birds exhibited rhythmic mean HMW (*p*<0.05, mesor ± SE: 0.45±0.06, respectively) levels of ADP in circulating plasma (Fig. 3A), suggesting that regulation of ADP concentration in plasma is accomplished using a biological clock. Greatest levels of total and HMW ADP occurred at Zeitgeber time (ZT: time since lights on) 3.5, and ZT 3.6, respectively, and lowest levels occurred at ZT 15.5, and ZT 15.6, respectively, according to the best fit cosinor function (Fig. 3). The average level of plasma HMW ADP approximately doubles from the migrating condition to the non-migrating condition. ADP intensity approximately doubled between peak and trough levels throughout the day. No significant circadian rhythm of average total and HMW ADP levels in migrating individuals (mesor ± SE: 0.45±0.03, 0.25±0.04, respectively) was detected, however, a sinusoidal trend was evident such that the peak levels of ADP in migrating birds appeared to be damped and phase-inverted compared with non-migrating birds, even though photoperiod was the same in both groups (Fig. 3). Plasma ADP levels of migrating birds were lowest during the day and greatest at night. Non-migrating birds also exhibited rhythmic expression of 136 kDa ADP (*p* = 0.03, mesor ± SE: 0.03±0.004) however, peak levels were estimated to be ZT 16.3, and the lowest levels at ZT 4.35, which is phase-inverted when compared with total and HMW ADP in non-migrating birds.

### Mixed-effects modeling of HMW adiponectin expression

Of the five mixed models considered, the best model contains only one fixed parameter: Migratory Status (Table 1). The evidence in support of this model was moderate (w = 0.55), with lower support for the Fat and Status + Fat models (w = 0.24, 0.20, respectively), and very little, or no support for the Interaction and Full models (w = 0.01, 0.00, respectively). Despite the appearance of a small absolute effect size on a log scale (β_1_ ± SE: −0.20±0.13), we consider migratory status to be an important factor in predicting HMW ADP levels, as a reduction of approximately half the relative amounts of HMW ADP in plasma of migrating birds is considerable. Our data demonstrate that a change in migratory disposition may underlie the complexity of the relationship between this fat hormone and body condition which may ultimately affect migratory behavior.

**Table 1:** Model selection results to predict plasma HMW adiponectin levels in the white-throated sparrow.

Model^a^	K_i_	AICc_i_	Δ_i_	w_i_
MigratoryStatus	5	9.61	0	0.55
Fat	5	11.21	1.60	0.24
MigratoryStatus + Fat	6	11.59	1.97	0.20
Interaction	7	17.38	7.77	0.01
Full	7	22.82	13.21	0.00

a *N* = 40; K is the number of parameters included in each model; AICc is the second order Akaike's information criteria; Δ_i_ is the difference between the AICc values of each model and the model with the lowest AICc; w_i_ is the weight of evidence for each model.

### Correlative analysis of plasma adiponectin and fat score

A strong positive correlation between ZT 6 HMW ADP and fat score was detected in migrating birds (HMW: r = 0.93; p<0.05). Strong (τ = −0.84) negative correlations were detected in 540 kDa ADP in migrating birds and in 136 kDa ADP of non-migrating birds (p = 0.05). We detected a negative correlation between fat score and the ratio of daytime to nighttime activity in migrating birds (τ = −0.79, p = 0.11) however, sample sizes were too small for statistical significance.

On average, non-migrating birds had a fat score 1 fat class lower than migrating birds (t-test: *p*<0.05; 95% confidence interval: −1.58, −0.44). We did not detect a sexual dimorphism in the average levels of plasma ADP, as has been observed in mammals [55], however, our sample of females was small (n = 3).

### Plasma visfatin

**Figure 4:**
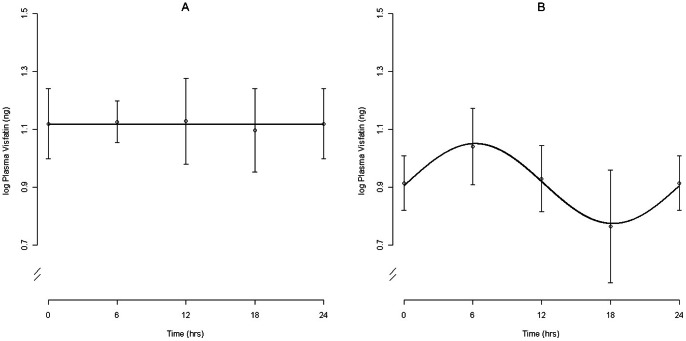
Expression of Visfatin. Levels of visfatin in plasma of non-migrating (A) and migrating (B) white-throated sparrows. For all birds, lights came on at ZT0 and went out at ZT 12. Migrating individuals as a group trended towards rhythmicity (*p*<0.10) with maximum concentrations at ZT6 and minimum concentrations at ZT18. Plasma levels in non-migrating birds were approximately 20% higher than those of migrating birds.

Plasma visfatin levels ranged from 0.34 ng/mL to 70.55 ng/mL (mean  = 12.01; med. = 9.37 ng/mL) (not log-transformed). We do not consider plasma visfatin rhythmic although, when values were averaged by time, migrating individuals as a group trended towards rhythmicity (*p*<0.10) with maximum concentrations at ZT6 and minimum concentrations at ZT18 (Figure 4).

Results from mixed-effects modeling of plasma visfatin are displayed in Table 2. Of the models considered, the best model contains only one fixed effect: Migratory Status. Evidence for this model was strong (w = 0.82), with less support for the Status + Fat model (w = 0.12) and little or no support for the remaining models. The Status model estimates a reduction of 0.62 ng/mL (SE = 0.18) (log scale) between the non-migrating and migrating groups, approximately a 20% reduction.

**Table 2:** Model selection results to predict plasma visfatin concentrations in the white-throated sparrow.

Model^a^	K_i_	AICc_i_	Δ_i_	w_i_
MigratoryStatus	5	131.31	0	0.82
MigratoryStatus+Fat	5	135.05	3.74	0.12
Full	6	137.78	6.47	0.03
Interaction	6	138.09	6.78	0.03
Fat	7	140.65	9.34	0.01
Sex + Fat	7	142.87	11.56	0.00

a *N* = 66; K is the number of parameters included in each model; AICc is the second order Akaike's information criteria; Δ_i_ is the difference between the AICc values of each model and the model with the lowest AICc; w_i_ is the weight of evidence for each model.

## Discussion

Our results demonstrate programmatic variations in the expression of the adipokines adiponectin and visfatin between the migratory and non-migratory conditions in white-throated sparrows. Adiponectin secretion is regulated by a circadian clock during non-migrating periods, and expression either becomes uncoupled from a circadian clock, or the rhythm of ADP secretion is damped and phase-inverted when birds are migrating. Although we do not know what aspect of the biological clockwork causes such regulatory changes in ADP, one may speculate that ADP output is a manifestation of changed clockwork in the adipose tissue of migrants. Although we did not detect a circadian rhythm of plasma visfatin concentration, we did observe a reduction in plasma visfatin during the migratory period. Because visfatin is able to communicate metabolic information to the molecular clock, a change in visfatin expression could modify the phase or amplitude of the biological clock. Our data therefore suggests a possible mechanism for regulating the changes in locomotor profiles observed during migration by modifying either ADP or visfatin expression, as plasma profiles of both of these adipokines changed between the non-migratory and migratory periods. Alternatively, ADP and visfatin levels could be altered more locally as part of the bird's overall preparation for increased metabolic demand needed during migration. For example, visfatin from skeletal muscle may act in a paracrine fashion to regulate glucose utilization locally. During migration, a birds' primary source of energy comes from the oxidation of adipose fatty acids [78]. Glucose is relatively unimportant as a fuel source in the long-distance endurance flights of migrating birds. In humans, visfatin has the ability to increase glucose uptake, playing a role in glucose homeostasis [79]. However, the seasonal decrease in plasma visfatin coinciding with the migratory period could reduce glucose utilization and allow for a greater reliance, on and utilization of, fat stores and fatty acids to fuel migration.

The shape of the circadian ADP profile approximates the daily profile of locomotor activity. In migrating birds of appropriate body condition, a low ADP profile during the day coincides with little daytime activity, and a higher ADP profile during the night coincides with the time of migratory flight. Conversely, the profile of ADP in diurnal, non-migrating birds is highest during the day, and lowest during the night. Compared to non-migrating birds, the phase shift in levels of ADP in migrating birds might be particularly adaptive for regulating lipid metabolism. Because adiponectin stimulates lipid metabolism without suppressing food intake [80], [81], it would be beneficial for migrating birds to increase ADP production at night. This hypothesis is supported in Fig. 3B, where it is demonstrated that birds signal to increase lipid metabolism during times of migratory flight. When migrating birds rest and refuel during the daytime, ADP levels are decreased, thereby allowing fat synthesis and preservation to occur until migratory flight continues [82]. In non-migrating birds, ADP levels are greatest during the day potentially allowing lipid metabolism to coincide with times of increased activity. Additionally, ADP levels are reduced at night when birds are sleeping, thereby preserving fat stores in non-migrating birds.

Correlations between HMW ADP and fat score, a proxy of body condition, in migrating birds are particularly intriguing because studies of mammals and chicken have demonstrated that levels of ADP decrease during obesity [77], [83], [84]. Our findings here demonstrate a positive correlation between fat and HMW ADP, the predominant ADP isoform in avian species. However, our scoring results were based solely on subcutaneous fat. The conflicting correlative findings regarding obese mammals and temporarily obese migrating birds may suggest that there are physiological differences between the genetically programmed, temporary subcutaneous fat stores maintained by birds to deal with the enormous energetic costs of migration and typical obesity in humans. In chicken, food deprivation leads to a significant decrease in ADP gene expression in adipose tissue [62], however, fasting has no effect on plasma ADP concentrations. Our results regarding non-migrating passerines are in opposition to these findings in chicken and demonstrate that plasma ADP is low at night when birds are not actively foraging (fasting), which could be a response to conserve fat stores until plasma ADP is high during the day, and food is available. Our results regarding migrating birds are similar, however, to those observed by Gomez-Abellan et al. [61], where the amplitude of ADP rhythms are attenuated in obese human subjects. Temporarily obese migrating white-throated sparrows display an average rhythm with a smaller amplitude compared with non-migrating individuals. Our results also agree with the findings of Calvani et al. [60], that plasma ADP concentrations increase after weight loss in human subjects. The circadian profile of non-migrating sparrows also matches the diurnal profile of normal-weight human subjects [60]. ADP is positively related to fat score which also displays a negative correlation with the ratio of daytime to nighttime activity, a relationship recently confirmed by Fusani et al. [85]. Migrating birds with low fat stores display greater daytime activity, perhaps foraging to replace lost fat, and migrating birds with greater fat stores spend more time night-active, displaying migratory restlessness.

Associations between sleep-wake cycles and weight regulation have been well documented, although the mechanisms linking the two processes are not well-understood [37], [86], [87]. If clocks in adipose tissue are able to communicate with clocks in the brain, in particular with the circadian clock controlling Zugunruhe [22], its disruption might account for the dramatically altered sleep-wake cycles associated with nocturnal migration. We suggest a role for clocks in adipose tissue regulating behavior, but further experimentation is needed to elucidate the mechanisms regulating peripheral oscillators in fat and the precise mechanisms regulating how they can influence behavior.

Our results provide evidence that a biological clock modulates the physiological transitions from non-migratory to nocturnal migratory behaviors. Adipokine signals may play a significant role in the initiation, continuation, and termination of nocturnal migration in small songbirds by indicating the amount of available fuel from fat and the overall body condition. Future experimentation utilizing restricted feeding schedules could be performed to force a “stopover” in captive birds during the migratory period. Analysis of plasma samples collected during forced stopovers could determine whether the change in the ADP profile observed in migrating versus non-migrating birds is applicable to stopover situations as well. Experiments to localize ADP receptors in the brain would also provide valuable information as to the specific location of neural substrates regulating the transition of behaviors in migratory birds. As this is the first study of the adipokines adiponectin and visfatin in migrant birds, further investigation of these and other adipokines in migrant species that store and use fat differently than the white-throated sparrow, such as shorter or long-distance migrants, would be relevant to a more complete understanding of the role of adipokines in metabolism and migration.

It is unclear from this study whether a change in adipokine signal drives a change in migratory disposition, influencing initiation or termination of migration or stopover delays, or a change in migratory status drives change in adipokine signals. However, because of adiponectin's close relationship with body fat, as well as migratory disposition, it is possible that ADP drives change in migratory disposition and changes in other adipokines such as visfatin make up the long list of adaptations necessary for successful migration.

## Materials and Methods

### Housing of animals

White-throated sparrows were captured in Centre County Pennsylvania during Spring 2008 using mist nets (USFWS #MB170276-0; PAGC #COL00194). Wild-caught sparrows were housed at the Poultry Education and Research Center (The Pennsylvania State University, University Park, PA) in a large, environmentally controlled indoor aviary. Birds were maintained under a 12 hr light, 12 hr dark photoperiod and were provided with food and water *ad libitum*. Birds were transferred to individual wire grated bird cages (9″×12″×15.5″, Top Wing®) in auditory contact with other birds and were allowed to acclimate for at least 2 weeks before experimentation began. All procedures were performed in accordance with standards approved by the Pennsylvania State University Institutional Animal Care and Use Committee (IACUC #27105, and #29985).

### Locomotor and behavioral activity

Infrared motion sensors (Quorum International) were mounted above each cage to record locomotor activity. Locomotor data were collected in 5 minute bins using the software program, VitalView (Mini Mitter, Respironics) and evaluated for daily activity with the software package, ActiView (Mini Mitter, Respironics). Migratory status was determined using the locomotor activity record by noting exclusively diurnal patterns of activity (non-migrating), and periods of excessive nighttime activity (migrating), as has been previously reported for WTSP [23], [24]. Nocturnal migratory restlessness is apparent in caged individuals as increased nocturnal activity, including flightless wing-whirring which corresponds to migratory flight in free-living birds. We used infrared video cameras (Q-See®) to continuously record the behaviors of individual birds.

We know that circannual program continued to run in these species based upon the presence of seasonal changes in molt, migratory restlessness, and reproductive behaviors and physiology (testicular recrudescence in males, and egg-laying in females during the summer months). Birds did experience temperature fluctuations across the seasons due to poor insulation in the building and it is possible that these seasonal temperature changes could provide seasonal cues to the birds. Temperatures ranged from approximately 17–28 Celsius across the year. Repeated changes in nocturnal activity observed in captivity approximated autumn and spring migratory periods of free-living conspecifics and indicate an endogenous control of migratory rhythms. In *Zonotrichia albicollis*, migratory behavior is expressed as the presence of nocturnal locomotor activity concomitant with little change in daytime activity [23], [24]. This is different than the locomotor profiles reported for other *Zonotrichia* species in captivity (i.e. a complete switch to exclusively nocturnal activity) [2], [20]. Changes in nocturnal activity were quantified using infrared motion sensors and video cameras were used to validate migratory disposition (Fig. 1 and Video S1) [22].

### Fat scoring

We utilized the subcutaneous fat score classification system developed by A. Kaiser [88], previously tested and validated by Soxhlet extraction in similar species of either the same order (Passiformes) or family (Emberizidae) [89]. This classification system has been shown to produce highly repeatable results by ranking fat scores from 0 (no fat) to 8 (extremely fat) by subcutaneous fat load in the furcular depression, breast muscles, and abdomen. Birds were evaluated bi-weekly, between the times of 3 and 4 ZT (ZT = zeitgeber time, time since lights on) to control for any daily changes in fat deposits. This method is widely practiced in field studies of small songbirds [90], [91], [92], [93].

### Western blot analysis of plasma ADP

Blood samples (∼50 µl) were collected from non-migrating (n = 5; fall, 2009) and migrating (n = 7; Winter/Spring 2010) birds from the brachial vein with heparinized capillary tubes. Two additional birds changed their migratory status during sampling and were excluded from the analysis. Samples were collected at four different times of day, in 6 hr intervals beginning with ZT0 (time of lights on) during one continuous autumn migratory period or non-migratory period. We allowed three days between each blood drawing for recovery. Samples were centrifuged at 2500 rpm for 20 minutes at 4°C and the plasma collected and stored until use at −80°C.

Using the Novex mini gel system and NativePAGE gels (Invitrogen®) we performed gel electrophoresis according to the manufacturer's instructions. Serial dilutions of plasma were performed to optimize the amount of protein loaded per well. A standard curve was generated from the signal intensity measures of seven different plasma dilutions to determine the linear range (r^2^ = 0.97) of the standard curve. The 1∶20 plasma dilution was chosen for further use from within the linear range of the standard curve. Plasma samples were prepared by adding 5 ul of 1∶20 diluted plasma sample to 4X Sample Buffer, G-250 Sample Additive, and deionized water. 10 µl of sample mixture was loaded into NativePAGE 4–16% Bis-Tris gel with NativeMark unstained (Invitrogen®), and Novex Sharp pre-stained (Invitrogen®) protein standards to provide a molecular mass reference, and a 1∶100 dilution of broiler chicken plasma was included as a positive control.

After separation, proteins were electrotransferred to a polyvinylidenedifluoride membrane. To check the uniformity of transfer, the gel was stained using Coomassie Blue. Membranes were incubated in SuperBlock Blocking Buffer (Pierce) for 1hr at room temperature, incubated in a custom generated primary antibody against a keyhole limpet hemocyanin conjugate in the N-terminal domain of chicken adiponectin (Rabbit anti-cADN; 1∶2500) [77], [94] overnight at 4°C, and incubated in secondary antibody (Pierce anti-rabbit IgG-HRP 1∶10,000) at room temperature for 1 hr. Enhanced chemiluminescence detection was performed on transparency film with ECL Plus Detection Reagent (Amersham) and imaged on the STORM 860 optical scanner using blue wavelength fluorescence. To determine the specific immunoreactivity of anti-adiponectin antibody, primary antibody was preadsorbed with chicken adiponectin protein [77].

### Enzyme-linked immunosorbant assay (ELISA) analysis of plasma visfatin

Blood samples (∼200 µl) were collected from the brachial vein using heparinized capillary tubes from non-migrating (n = 9) and spring migrating (n = 8) individuals. Samples were collected at four different times of day (ZT 0, 6, 12, 18) to assess circadian rhythmicity. We allowed at least 2 days between sampling for the birds to recover. Blood samples were centrifuged at 2500 rpm for 20 minutes at 4°C and the plasma was stored until use at −80°C. Two plasma samples were found to have inadequate volume and were discarded from the analysis.

Undiluted plasma visfatin concentrations were determined by ELISA (Phoenix Pharmaceuticals, Burlingame, CA, Visfatin C-Terminal (Human) Kit), following the manufacturer's instructions. The use of this kit for detecting avian plasma visfatin was previously validated in chicken [69]. Samples and controls were assayed in duplicate using two plates (intra-assay variation: plate 1 = 11.6%; plate 2 = 5.1%). Samples from both migrating and non-migrating birds were interspersed on both plates as a safeguard against nondemonic intrusion. Samples were read at 450 nm using the FLUOstar Omega microplate reader; standard curves were generated using 4-parameter logistics based on blank corrected averages of duplicates. Sample volumes of one time point in two individuals were inadequate and not used for analysis.

### Data analysis

The ECL signal intensity of ADP bands from Western blots was quantified (Image Quant 5.1) in arbitrary intensity units. Protein amount was determined by Bradford assay and signal intensity was normalized to individual protein concentrations. A calibration curve was constructed for each Bradford protein assay using five dilutions of bovine serum albumin in triplicate, including water blanks, using Bio-Rad Protein Assay Dye Reagent Concentrate. Sparrow plasma samples were diluted 1∶100 to fall within the linear range of BSA standard curves and assayed in duplicate. Total plasma adiponectin levels were quantified and additional analyses were performed to quantify individual multimeric bands. Assumptions of homoscedacity and normality were assessed by evaluating normal probability plots. Non-normally distributed samples were log_10_ transformed to accommodate this assumption; bands that did not meet the normality assumption after transformation were analyzed using nonparametric statistics (Kendall tau rank correlation). Average total ADP (sum intensity of all isoforms), individual isoforms of ADP, and visfatin concentrations were analyzed using linear harmonic regression in cosinor analysis using the program CircWave [95] to determine the presence of a circadian rhythm.

To accommodate dependency among levels of hormone sampled over a short time series, we employed a mixed modeling approach to examine the importance of migratory status (migrating vs. non-migrating), time of day (ZT 0, 6, 12, 18), sex (visfatin data only) and fat score in predicting hormone levels. To identify the most important variables in predicting levels of plasma HMW ADP, we compared five linear mixed-effects models using second order Akaike information criteria (AICc) to correct for small sample sizes, fitted in the software program “R” 2.10.1 [96] which is based on log-likelihood and the number of model parameters to quantify the evidence of support for each proposed model. The AICc value provides a measure of distance between competing models and allows for model comparison. Akaike weights are similar to probabilities and describe the weight of evidence in support for each model in minimizing the distance from the “true model” and allows for comparison of evidence for each model in a group of models. This weight can be interpreted as the relative probability of the model given the data [97], [98]. A model with an Akaike weight approaching 1 is unambiguously supported by the observed data [99]. We began by considering a Full model (“Full”), including all three explanatory variables. We dropped the least important variable (time) and considered a model with migratory status and fat (“Status + Fat”), and a model with an interaction between group and fat (“Interaction”). Lastly, we considered models with migratory status (“Status”) and fat (“Fat”) individually. To account for the effects of autocorrelation in repeated sampling over time, we used an AR1 error structure and included “individual” as a random effect. The model with the smallest relative AICc value indicates the best model of those proposed. Residuals were examined to assess homogeneity and verify normality. Examination of residuals versus fitted values ensured that the model fit our data well.

Six mixed-effects models were evaluated to predict plasma visfatin in white-throated sparrows. Migratory status, fat score, and sex, were included as fixed effects while individual and time of day were included as random effects. We used time as a random effect because plasma visfatin did not display circadian rhythmicity. We considered a Full model (“Full”), including all three fixed effects, an interaction model between migratory status and fat (“Interaction”), an additive model of migratory status and fat (“Status + Fat”), an additive model of sex and fat (“Sex + Fat”), as well as models with migratory status (“Status”) and fat (“Fat”) alone. Residual plots were used to evaluate fit of the models.

We calculated correlations between circulating plasma ADP isoform levels and fat score, and the ratio of daytime to nighttime activity and fat score with “R”. We calculated this activity ratio rather than raw nighttime activity values to correct for individuals with varying baseline activity levels. Data for correlations of ADP and fat score were grouped by migratory disposition (exhibiting migratory restlessness or not) and ADP levels were evaluated at ZT 6 (the time closest to when fat scores were collected). Correlations between fat score and activity ratio were performed based on values averaged by individual. Because the fat classification system used in this study has a relatively large category size, and the distribution of ordinally measured fat scores is normal, ordinal fat score values were treated as continuous [100], [101].

## Supporting Information

Video S1 **Shown is a segment of a video recording taken during the night under infrared illumination.** Video images show intense activity, including wing-whirring, features which were used to determine whether a bird was exhibiting nocturnal migratory restlessness.
(WMV)
